# Molecular insights into glioblastoma progression: role of CHCHD2P9 in tumor heterogeneity and prognosis

**DOI:** 10.3389/fimmu.2025.1581850

**Published:** 2025-06-24

**Authors:** Yanyu Ding, Lizhi Xiao, Xiaoling Zhou, Jiaxin Zhao, Jianli Ke, Huabao Cai, Mengyu Zhao, Cunzhi Wang, Tianhang Yu, Zhijie Zhao, Yucai Wang, Jiyuan Ke

**Affiliations:** ^1^ Department of Immunology, School of Basic Medical Sciences, Center for Big Data and Population Health of Institute of Health and Medicine (IHM), Anhui Medical University, Hefei, China; ^2^ Institute of Health and Medicine, Hefei Comprehensive National Science Center, Hefei Economic and Technological Development Zone, Hefei, Anhui, China; ^3^ Department of Neurosurgery, The First Affiliated Hospital of Anhui Medical University, Anhui Medical University, Hefei, China; ^4^ Department of Plastic and Reconstructive Surgery, Shanghai Ninth People’s Hospital, Shanghai JiaoTong University School of Medicine, Shanghai, China; ^5^ The Chinese Academy of Sciences (CAS) Key Laboratory of Innate Immunity and Chronic Disease, School of Basic Medical Sciences, Division of Life Sciences and Medicine, University of Science and Technology of China, Hefei, Anhui, China

**Keywords:** glioblastoma, CHCHD2P9, diagnosis, prognosis, central nervous system, tumor microenvironment

## Abstract

**Background:**

Gliomas are highly aggressive, life-threatening tumors with poor prognosis, and remain a leading cause of mortality among brain cancers. Although the role of mitochondrial proteins in cancer has garnered increasing attention, their specific functions in the nervous system, particularly in gliomas, remain poorly understood.

**Methods:**

We integrated single-cell RNA sequencing with cellular assays and flow cytometry to investigate the molecular characteristics and cellular interactions within glioblastoma subpopulations during tumor progression.

**Results:**

Single-cell RNA sequencing revealed several differentially expressed genes (DEGs) within glioblastoma subpopulations. Trajectory analysis identified CHCHD2P9 as a pivotal marker for the terminal subpopulation. Moreover, elevated expression of CHCHD2P9 was found to correlate with poorer clinical outcomes. Subsequent cellular experiments further explored the underlying mechanisms driving these observations.

**Conclusions:**

CHCHD2P9 is significantly overexpressed in glioma patients, and its differential expression plays a crucial role in regulating glioma cell proliferation and migration. A CHCHD2P9-based risk model holds promise as both a prognostic biomarker and a potential therapeutic target, providing novel insights into the pathogenesis of gliomas and opening avenues for personalized treatment strategies.

## 1 Introduction

Glioblastoma multiforme (GBM) represents the most prevalent and aggressive subtype of primary brain malignancies, comprising approximately 50% of all glioma cases and presenting substantial challenges in both diagnosis and therapeutic management. Epidemiological data indicate a global annual incidence of 4 to 5 cases per 100,000 individuals, with age-adjusted incidence rates exhibiting regional variability (1, 2). Epidemiological data indicate a global incidence of 4–5 gliomas per 100,000 individuals annually, with age-adjusted rates (3, 4). Histologically, GBM is characterized by substantial cellular heterogeneity, rapid mitotic activity, and infiltrative growth patterns, all of which contribute to its complexity and resistance to conventional therapies (5, 6). The standard treatment for GBM—comprising surgical resection, radiation therapy, and chemotherapy—remains largely ineffective in preventing tumor recurrence and improving long-term survival, with median survival after diagnosis typically less than 24 months. The molecular underpinnings of GBM involve extensive dysregulation of key signaling pathways, including those governing cell proliferation, apoptosis, and DNA repair, which are further compounded by chromosomal instability (7). Despite the advent of innovative therapies, such as immune checkpoint inhibitors, targeted molecular therapies, and gene-editing strategies, their clinical application is still in the experimental phase, hindered by challenges including the selective permeability of the blood-brain barrier (BBB) and the lack of reliable prognostic biomarkers. The urgent need for more effective treatments highlights the importance of further elucidating the molecular and immune landscape of gliomas, as well as identifying novel therapeutic targets that can overcome existing treatment barriers (8, 9).

Mitochondrial dysfunction is increasingly recognized as a hallmark of both neurodegenerative diseases and cancer. Members of the CHCHD gene family, such as CHCHD2 and CHCHD10, encode mitochondrial proteins that play important roles in regulating cellular energy homeostasis, oxidative stress responses, and apoptosis (10, 11). Mitochondria, as the powerhouse of the cell, are crucial for maintaining cellular homeostasis, including energy production, oxidative stress response, and apoptosis regulation (12, 13). CHCHD2P9, annotated as a pseudogene (coiled-coil-helix-coiled-coil-helix domain containing 2 pseudogene 9), shares high sequence similarity with CHCHD2 and is predicted to contain similar structural domains. However, current databases such as UniProt and GeneCards do not provide definitive evidence that CHCHD2P9 encodes a functional protein, and its role remains largely uncharacterized in both physiological and pathological contexts.

Some studies have reported RNA expression of CHCHD2P9 in specific tissues, suggesting that it may exert biological effects through non-coding RNA mechanisms or through its sequence homology with protein-coding genes. Recent bioinformatic analyses and emerging evidence suggest potential involvement of CHCHD2P9 in processes such as neuronal differentiation, synaptic plasticity, and neuroinflammatory responses. These observations, though preliminary, raise the possibility that CHCHD2P9 may modulate neurodegenerative processes, either through RNA-mediated regulation or inferred protein-like activity.

Furthermore, mitochondrial dysfunction—a common feature in gliomas—is often driven by altered expression of mitochondrial-related genes. While the role of CHCHD2 and related proteins in tumorigenesis has been more clearly elucidated, the functional contribution of CHCHD2P9 in glioma remains poorly understood. Given its putative mitochondrial association and sequence homology, CHCHD2P9 warrants further investigation as a potentially novel regulatory molecule in glioma biology, although current evidence does not yet support its classification as a bona fide mitochondrial protein (14).

The advent of high-resolution single-cell RNA sequencing (scRNA-seq) has fundamentally transformed the study of cellular heterogeneity, enabling detailed characterization of individual cell states and their interactions in both physiological and pathological settings (15, 16). This technology offers unparalleled resolution in identifying cellular subpopulations within tumors, thereby providing critical insights into the molecular underpinnings of glioma progression, including mitochondrial dysfunction and metabolic reprogramming (17, 18). Moreover, scRNA-seq facilitates the in-depth analysis of complex intercellular communication networks, particularly those involving tumor cells, immune infiltrates, and neurons within the glioma microenvironment. These investigations have shed light on key neuroimmunological interactions that influence tumor behavior, such as immune modulation by tumor-associated immune cells and neuroimmune crosstalk contributing to glioma invasiveness (19, 20). The integration of single-cell transcriptomics with spatial transcriptomics further enhances this approach by allowing researchers to construct high-resolution tumor atlases. These atlases delineate the spatial architecture of cellular clusters and their associated signaling pathways, elucidating critical drivers of tumor growth and invasion (21, 22). Beyond mechanistic discoveries, single-cell multiomic technologies are poised to revolutionize glioma treatment strategies. By enabling the identification of tumor-specific neoantigens, immune checkpoint signatures, and other predictive biomarkers, these platforms can inform the development of personalized immunotherapeutic interventions (23, 24). Notably, scRNA-seq permits the precise identification of immune cell subsets—including tumor-associated macrophages, T lymphocytes, and dendritic cells—whose interactions with tumor cells are pivotal to the success of immunotherapies. These insights into the tumor-immune interface are essential for the advancement of precision oncology, particularly in tailoring immunotherapeutic approaches to target specific glioma subtypes or overcome immune evasion mechanisms (25). Collectively, the integration of single-cell technologies with immunological profiling provides a comprehensive framework for dissecting glioma biology and paves the way for next-generation, precision-driven therapeutic strategies.

## 2 Methods

### 2.1 Data source

The single-nucleus RNA sequencing (snRNA-seq) data used in this study were obtained from the Gene Expression Omnibus (GEO) database (https://www.ncbi.nlm.nih.gov/geo/) under accession number GSE103224 (26). The dataset includes specific samples, namely GSM2758471, GSM2758472, GSM2758473, GSM2758474, GSM2758476, GSM2758477, and GSM2940098. Additionally, RNA sequencing data for various cancer types were retrieved from The Cancer Genome Atlas (TCGA) through the Genomic Data Commons (GDC) portal (https://portal.gdc.cancer.gov/). These datasets were integrated for single-cell bioinformatics analyses to assess gene expression profiles and characterize cellular diversity, with a particular focus on glioma.

### 2.2 Data standardization and quality control

The single-cell RNA sequencing (scRNA-seq) data were processed and analyzed using the Seurat package (v4.3.0) within the R programming environment (v4.2.0). Rigorous quality control (QC) procedures were implemented to ensure high-quality data and minimize noise from low-quality cells or potential doublets. DoubletFinder (v2.0.3) was used to detect and exclude potential doublets, and the following filtering criteria were applied: number of detected genes (nFeature) between 300 and 4,500, total read counts (nCount) between 500 and 100,000, and mitochondrial gene expression fraction less than 5%. These thresholds were designed to eliminate low-quality or contaminated cells (27).

For the bulk RNA-seq analysis, gene expression profiles from The Cancer Genome Atlas (TCGA) and Chinese Glioma Genome Atlas (CGGA) were included. Raw count and normalized expression data were downloaded, and samples without complete clinical information were excluded. To address technical variation and platform differences between datasets—particularly those arising from RNA-seq and microarray platforms—batch effect correction was conducted using the sva package’s ComBat function (v3.44.0), which is specifically suited for cross-platform integration. Data were log2-transformed and normalized prior to downstream analysis.

Following quality control, the single-cell expression matrix was normalized using Seurat’s default method to account for differences in sequencing depth and capture efficiency. The top 2,000 highly variable genes (HVGs) were identified and retained for subsequent analyses. Harmony (v0.1.1) was used for further batch effect removal across different single-cell experimental conditions, thereby enhancing the biological comparability of the samples.

Dimensionality reduction was performed using principal component analysis (PCA), and the top 30 principal components (PCs) were selected to represent the major sources of variation in the dataset. Uniform Manifold Approximation and Projection (UMAP) was applied to visualize cellular heterogeneity in two-dimensional space.

Cell type annotation was conducted based on known marker genes retrieved from the literature and the CellMarker database (http://xteam.xbio.top/CellMarker/). Cell clustering was subsequently performed, and the proportion of each annotated cell population was quantified and visualized 282828.

To explore the clinical significance of CHCHD2P9, glioma patients from both TCGA and CGGA datasets were stratified into high and low CHCHD2P9 expression groups based on the median expression value. This median-based cutoff ensures robust group sizes and is commonly used in survival and clinical correlation studies. The clinical relevance of this grouping strategy was further assessed through Kaplan–Meier survival analysis and Cox regression to confirm its prognostic value (28).

### 2.3 Identification of differentially expressed genes and functional pathway analysis

Differentially expressed genes (DEGs) for each cell type were identified using Seurat’s “FindAllMarkers” function. The Wilcoxon rank-sum test was employed to compare gene expression profiles across different clusters, as it is well-suited for non-parametric analysis of non-normalized expression data. The DEGs were selected based on two criteria: (1) a minimum log fold change (logFC) of 0.25 to ensure meaningful differences in expression levels between groups, and (2) the expression of the gene in at least 25% of the cells in the respective cluster, ensuring that the selected genes were sufficiently prevalent within the population.

To further explore the biological relevance and associated pathways of the DEGs, functional enrichment analysis was conducted using the “clusterProfiler” R package (version 0.1.1). This analysis provided insights into the enriched biological pathways, aiding in the interpretation of the functional roles of the DEGs in the context of the tissue or disease under study. The enrichment results were visualized through dot plots and bar charts for a more intuitive representation of the biological processes and signaling pathways involved.

### 2.4 Visualization of cell clusters and subpopulations

To investigate cellular heterogeneity within glioma, a comprehensive analysis of glioma cells was performed. The raw data were normalized to correct for sequencing depth discrepancies, ensuring comparability across samples. After normalization, the 2,000 most variable genes (HVGs) were selected to represent the highest variability in expression across the glioma cell population. These genes were subsequently standardized to ensure consistent scaling for further analyses.

The Harmony algorithm (v0.1.1) was applied to address potential batch effects from sample collection or processing, integrating data from multiple batches while preserving biological differences. PCA was performed, retaining the top 30 principal components (PCs) that captured the majority of variance in the dataset. These PCs were then used for clustering analysis to group cells based on shared gene expression profiles, facilitating the identification of unique subpopulations within the glioma cell population.

UMAP, a non-linear dimensionality reduction technique, was applied to visualize the cellular diversity and identify distinct cell clusters, reflecting different functional or phenotypic states within the glioma tumor microenvironment.

### 2.5 Identification of malignant cells using inferCNV

Copy number variation (CNV) analysis was performed to distinguish malignant tumor cells from non-tumor cells using the inferCNV tool. Vascular endothelial cells were selected as the reference group due to their genomic stability in glioma. CNV profiles were generated for each cell subpopulation to identify chromosomal aberrations indicative of malignant transformation.

Subpopulations with significant deviations from the reference CNV profile, particularly those with focal amplifications or deletions, were classified as glioblastoma (GBM) cells. These GBM cells were further analyzed to examine their genomic instability and their potential association with other cellular features such as gene expression patterns and phenotypic traits, providing a comprehensive understanding of the malignant glioma subpopulation.

### 2.6 Differential gene expression of subpopulations

The differential gene expression across different glioma cell subpopulations was assessed using Seurat’s “FindAllMarkers” function. Genes with significant differential expression were selected based on fold change and statistical significance, and these genes were further analyzed to explore their functional roles.

### 2.7 Investigating differentiation pathways in glioma cell subpopulations

To comprehensively characterize the differentiation trajectories and dynamic phenotypic transitions among glioma cell subpopulations, an integrative approach utilizing three independent computational tools was employed.

Initially, cytoTRACE was applied to infer the differentiation potential and stemness of individual cells based on gene expression entropy and transcriptional diversity. This algorithm estimates a stemness score for each cell by evaluating genes associated with undifferentiated cellular states, allowing for the hierarchical positioning of glioma subpopulations along a continuum from stem-like to more differentiated phenotypes.

Subsequently, Monocle2 (v2.24.0) was utilized to reconstruct single-cell developmental trajectories using the DDRTree dimensionality reduction method. Variable genes with high expression dispersion across cells were identified using the FindVariableFeatures function to serve as input for trajectory construction. Pseudotime analysis was conducted to model the temporal progression of cellular states, enabling the visualization of differentiation pathways and identification of branch points representing potential lineage bifurcations.

To further refine lineage relationships, Slingshot (v2.6.0) was applied to the reduced-dimensional embedding of glioma subpopulations. A minimum spanning tree (MST) framework was employed to define global connectivity among cellular clusters. The getLineages function was used to infer developmental lineages and pseudotemporal ordering, facilitating the mapping of putative differentiation paths and lineage hierarchies within the tumor ecosystem.

This integrative strategy combining cytoTRACE, Monocle2, and Slingshot provided robust insights into the differentiation dynamics, lineage commitment, and phenotypic heterogeneity of glioma cell subpopulations.

### 2.8 Cell communication

To investigate intercellular communication within the glioblastoma tumor microenvironment, the CellChat R package (v1.6.1) was employed. This analysis identified key receptor-ligand interactions across different cell populations, providing insights into how tumor and stromal cells coordinate signaling activities. The resulting communication networks were analyzed to uncover patterns of intercellular signaling, contributing to understanding tumor progression, immune evasion, and microenvironment remodeling in glioblastoma.

Potential therapeutic targets for modulating intercellular communication were identified by analyzing the intensity and direction of signaling between cell populations.

### 2.9 Development and assessment of prognostic models

To identify potential prognostic biomarkers for glioma, marker genes obtained from glioma cohorts were subjected to univariate survival analysis using the “survival” R package. This analysis assessed the relationship between gene expression patterns and patient survival outcomes.

Following univariate analysis, Lasso regression was used to select genes with the highest predictive value, reducing model complexity and preventing overfitting. These genes were then incorporated into a multivariate Cox proportional hazards regression model, evaluating their collective prognostic significance.

The risk score for each patient was computed using the formula:

RiskScore=(Gene_1_​×Coefficient_1_​)+(Gene_2_​×Coefficient_2_​)+…+(Gene_n​_×Coefficient_n​_).

Patients were stratified into high- and low-risk groups based on the median risk score, and overall survival differences between groups were assessed using Kaplan–Meier survival curves and the log-rank test. To validate the proportional hazards (PH) assumption underlying the Cox model, Schoenfeld residuals were examined using the cox.zph function in the survival package (v3.5-5). No significant violation of the PH assumption was observed.

To evaluate the independent prognostic value of the risk model, a multivariate Cox proportional hazards regression analysis was performed, incorporating established clinical covariates, including patient age, WHO tumor grade, IDH mutation status, and MGMT promoter methylation status. These variables were selected based on their known relevance to glioma prognosis. The adjusted hazard ratios (HRs) and corresponding 95% confidence intervals (CIs) were reported.

In addition, the prognostic accuracy of the model was assessed using time-dependent receiver operating characteristic (ROC) curves, and the area under the curve (AUC) was calculated at 1-, 3-, and 5-year survival endpoints to evaluate the model’s predictive performance.

### 2.10 Immune cell infiltration in the tumor microenvironment

Immune cell infiltration within the glioma tumor microenvironment was comprehensively evaluated using multiple computational algorithms to ensure methodological robustness and minimize tool-specific bias. Specifically, CIBERSORT (v1.06), TIMER2.0, xCell, and ESTIMATE (v1.0.13) were applied to infer the relative abundance of 22 immune cell types and stromal components across tumor samples. The results derived from these distinct tools were cross-compared to enhance the reliability of immune composition estimates. The correlations between immune cell infiltration patterns and CHCHD2P9 expression levels, as well as overall patient prognosis, were systematically analyzed.

To investigate mechanisms of immune evasion, the Tumor Immune Dysfunction and Exclusion (TIDE) algorithm was also employed, generating TIDE scores for each sample to quantify the degree of immune dysfunction and exclusion within the tumor microenvironment. Comparisons of immune infiltration and immune evasion scores were conducted between high- and low-risk groups stratified by the prognostic model.

Furthermore, to complement the GO and KEGG enrichment results, Gene Set Enrichment Analysis (GSEA, v4.3.2) was performed using the “clusterProfiler” R package (v4.6.2) to identify enriched biological pathways without relying on arbitrary differential expression thresholds. This unbiased approach provided additional insight into the biological processes associated with CHCHD2P9 expression.

### 2.11 Differential expression and functional enrichment analysis in bulk data

To identify differentially expressed genes (DEGs) between high-risk and low-risk glioma groups, gene expression analysis was performed using the DESeq2 package in R. DEGs were identified with the following criteria: |logFC| > 2 and a p-value < 0.05, allowing for the detection of genes with significant expression changes across the two risk groups. DESeq2 employs a negative binomial distribution to model RNA-sequencing data, ensuring robust statistical analysis for differential gene expression.

Subsequent to DEG identification, functional enrichment analyses were conducted to explore the biological significance of these genes. Gene Ontology (GO) analysis categorized the DEGs into three major functional domains: biological processes (BP), molecular functions (MF), and cellular components (CC). Additionally, Kyoto Encyclopedia of Genes and Genomes (KEGG) pathway analysis was performed to identify the key molecular pathways involved, thus providing a more comprehensive understanding of the biological changes in high-risk glioma patients compared to low-risk counterparts.

Gene Set Enrichment Analysis (GSEA) was also employed to explore the coordinated expression of gene sets associated with pre-defined biological processes or pathways. GSEA identifies pathways that are differentially activated or repressed between risk groups, even when individual genes do not show significant expression changes.

These combined enrichment analyses facilitated the identification of critical biological pathways and functional categories contributing to glioma progression and risk stratification, shedding light on the molecular mechanisms underlying glioma and providing potential targets for novel therapeutic strategies.

### 2.12 Somatic mutation analysis

To assess the mutational landscape of gliomas, somatic mutation data were retrieved from The Cancer Genome Atlas (TCGA) database. The focus of the analysis was on frequently mutated genes and those included in the risk stratification model, with the aim of identifying recurrent mutations and exploring their functional relevance.

The tumor mutational burden (TMB) was calculated for each glioma sample using the “maftools” R package, a tool specifically designed for processing and visualizing cancer genomic data. TMB was defined as the number of non-synonymous mutations per megabase of the genome, which serves as an important metric for characterizing the mutational profile of individual tumors. Glioma samples were subsequently categorized into high-TMB and low-TMB subgroups based on the median TMB value to investigate potential correlations with clinical outcomes.

Kaplan-Meier survival curves were constructed to compare overall survival (OS) between high-TMB and low-TMB groups, and statistical significance was assessed using log-rank tests, providing insights into the potential of TMB as a prognostic biomarker in glioma.

Furthermore, copy number variation (CNV) profiles of the model genes were analyzed to identify genomic alterations associated with glioma progression. CNV analysis was performed to detect amplifications and deletions in genes of interest, further elucidating the genomic instability driving glioma development. The relationship between CNV alterations and patient prognosis was also examined to explore their potential impact on tumor progression and clinical outcomes.

### 2.13 Drug sensitivity analysis

To evaluate the drug sensitivity of glioma samples, the pRRophetic R package (version 0.5) was used to predict drug responses based on gene expression data. The half-maximal inhibitory concentration (IC50) for a range of chemotherapy drugs was computed for each glioma sample. IC50 values, which represent the drug concentration required to inhibit 50% of cell viability, provide a crucial measure of drug efficacy.

IC50 values were calculated for multiple commonly used chemotherapy agents, enabling the profiling of tumor responsiveness to various drugs. This approach allowed for the identification of glioma samples with differing sensitivities to specific chemotherapeutic compounds, contributing to a better understanding of drug efficacy within the cohort.

The drug sensitivity data were further analyzed in conjunction with molecular features of the tumors, including gene expression profiles and mutation statuses. This analysis explored potential associations between molecular characteristics and chemotherapy response, facilitating the identification of drugs that could be more effective in specific glioma subgroups, thus paving the way for personalized treatment strategies based on tumor molecular profiling.

### 2.14 Cell culture

The U-87 MG (human glioblastoma) and LN229 (human glioma) cell lines were obtained from ATCC (Manassas, VA, USA). Cells were cultured in high-glucose DMEM (Gibco, Thermo Fisher Scientific, Waltham, MA, USA) supplemented with 10% fetal bovine serum (FBS) and 1% penicillin-streptomycin (both Gibco, Thermo Fisher Scientific, Waltham, MA, USA). Cultures were maintained at 37°C in a humidified 5% CO_2_ atmosphere.

For subculture, cells were passaged when they reached approximately 80% confluence. This was achieved by trypsinization with 0.25% trypsin-EDTA (Gibco, Thermo Fisher Scientific, Waltham, MA, USA). The resulting cell suspensions were diluted with fresh culture medium and subcultured at a 1:3 ratio.

For experiments, cells were seeded in 6-well (Corning, Corning, NY, USA) or 96-well (Corning, Corning, NY, USA) plates at appropriate densities based on the specific experimental requirements. The culture medium was refreshed every 2–3 days, and all experiments were conducted using cells within passage numbers 3–15.

### 2.15 Cell transfection

To specifically knock down CHCHD2P9 expression, two short hairpin RNAs (shRNAs) were designed and synthesized by Ribobio (Guangzhou, China). The shRNAs were selected for high efficiency and specificity. Transfection of shRNAs was performed using Lipofectamine 3000 (Invitrogen, Thermo Fisher Scientific, Waltham, MA, USA), following the manufacturer’s guidelines.

Briefly, Lipofectamine 3000 and shRNA plasmid were combined in Opti-MEM medium (Gibco, Thermo Fisher Scientific, Waltham, MA, USA) and incubated at room temperature for 20 minutes to form transfection complexes. These complexes were introduced into cells cultured in complete medium. Cells were then incubated for 6 hours at 37°C in a 5% CO_2_ incubator. After incubation, the transfection mixture was replaced with fresh culture medium. The effectiveness of gene knockdown was evaluated 48–72 hours post-transfection by RNA extraction and subsequent analysis.

### 2.16 RNA extraction and quantitative PCR analysis

Total RNA was extracted from cultured cells using TRIzol reagent (Thermo Fisher Scientific, Cat. No. 15596018), following the manufacturer’s protocol. RNA was separated by chloroform extraction and precipitated with isopropanol. The RNA pellet was washed with 75% ethanol and resuspended in RNase-free water. RNA concentration and purity were determined using a NanoDrop spectrophotometer (Thermo Fisher Scientific, Wilmington, DE, USA), and RNA integrity was confirmed via agarose gel electrophoresis.

For complementary DNA (cDNA) synthesis, 1 μg of total RNA was reverse-transcribed using the PrimeScript™ RT Reagent Kit (Vazyme Biotech, Nanjing, China, Cat. No. R232-01), following the manufacturer’s instructions. The reverse transcription reaction was performed in a 20 μL volume, with an incubation at 37°C for 15 minutes, followed by 85°C for 5 seconds to terminate the reaction.

Quantitative real-time PCR (qPCR) was performed using SYBR Green qPCR Master Mix (Vazyme Biotech, Cat. No. Q111-02) on the Roche LightCycler 480 System (Roche Diagnostics, Mannheim, Germany). PCR conditions included an initial denaturation at 95°C for 10 minutes, followed by 40 cycles of 95°C for 15 seconds and 60°C for 30 seconds. A melting curve analysis was conducted to confirm the specificity of the amplified products.

Relative gene expression was calculated using the 2−ΔΔCt method, with β-Actin as the internal control gene. Primer sequences for target genes and β-Actin were custom-designed and synthesized by Tsingke Biotech (Beijing, China), and are listed in **Supplementary Table 1**.

### 2.17 Cell viability assay

Cell viability was assessed using the Cell Counting Kit-8 (CCK-8) assay (Vazyme Biotech, Nanjing, China, Cat. No. A311-01). Cells were seeded into 96-well plates at a density of 1 × 10³ cells/well, with a final volume of 100 μL per well. Following a 24-hour incubation at 37°C in a 5% CO_2_ atmosphere, 10 μL of CCK-8 reagent was added to each well, and cells were incubated at 37°C for 2 hours.

Cell viability was determined by measuring absorbance at 450 nm using a microplate reader (Thermo Fisher Scientific, Waltham, MA, USA). Absorbance was measured at 0, 24, 48, 72, and 96 hours, allowing for the construction of a cell growth curve to analyze proliferation dynamics.

### 2.18 Colony formation assay

To evaluate the colony-forming ability of cells, a total of 1000 cells were seeded in each well of 6-well plates and cultured for approximately 14 days to allow colony formation. Cells were maintained in complete culture medium, which was replaced every 2–3 days. After the 2-week incubation period, colonies were examined using a light microscope at low magnification to assess the presence of visible cell clusters.

Following visual inspection, the cells were gently washed with phosphate-buffered saline (PBS) to remove any residual culture medium. The colonies were then fixed with 4% paraformaldehyde (PFA; Solarbio, Beijing, China) at room temperature for 15 minutes. After fixation, colonies were stained with a 0.1% crystal violet solution (Solarbio, Beijing, China) for 20 minutes at room temperature to enhance visibility. Excess dye was removed by washing with PBS, and the samples were air-dried at room temperature.

For colony quantification, crystal violet-stained colonies were manually counted. Only colonies containing 50 or more cells were included in the count. The colony count per well was recorded, and data were expressed as the mean ± standard deviation (SD) from at least three independent experiments.

### 2.19 Wound healing assay (scratch assay)

After transfection, cells were seeded into 6-well plates and cultured in complete medium until they reached approximately 95% confluency. A uniform linear scratch was created in the cell monolayer using a sterile 200 µL pipette tip. Following the scratch, the wells were gently rinsed twice with phosphate-buffered saline (PBS) to remove any dislodged cells and debris. The cells were then maintained in serum-free medium to prevent cell proliferation during the wound healing process.

The cells were incubated in serum-free medium for 48 hours at 37°C in a 5% CO_2_ incubator. Wound area images were captured at specific locations immediately after wounding (0 hours) and after 48 hours of incubation using a phase contrast microscope (model). To ensure consistency, images were taken from the same locations at both time points.

The width of the scratch was measured using ImageJ software (National Institutes of Health, Bethesda, MD, USA). The percentage of wound closure was calculated using the formula:

Percentage of wound closure =(Scratch width at 0hScratch width at 0h−Scratch width at 48h)​×100%.

The results were expressed as the mean ± standard deviation (SD) from a minimum of three independent experiments.

### 2.20 Transwell migration assay

Cell migration was assessed using Transwell chambers (Corning, Corning, NY, USA). For each assay, 2 × 10^4^ cells in 200 µL of serum-free medium were seeded into the upper compartment of the Transwell chamber. To assess extracellular matrix (ECM)-dependent migration, the upper chamber was pretreated with or without Matrigel (BD Biosciences, Franklin Lakes, NJ, USA) according to the experimental design. Matrigel (100 µg/mL) was applied and polymerized at 37°C for 1 hour, while control wells were left untreated.

The Transwell chambers were incubated for 48 hours at 37°C in a 5% CO_2_ incubator, with a chemotactic gradient established by adding medium containing 10% fetal bovine serum (FBS) to the lower chamber. After incubation, non-migratory cells retained on the upper surface of the membrane were gently removed using a cotton swab.

Cells that migrated to the lower surface of the membrane were fixed with 4% paraformaldehyde (PFA, Solarbio, China) for 15 minutes at room temperature, followed by staining with 0.1% crystal violet (Solarbio, China) for 20 minutes. The membranes were rinsed with PBS to remove residual dye and air-dried at room temperature.

The migrated cells were quantified by counting the crystal violet-positive cells in five randomly captured fields under a light microscope (model). Representative images were captured for documentation. Migration capacity was expressed as the average number of cells per field, and results from three independent experiments were reported as the mean ± standard deviation (SD).

### 2.21 Assessment of apoptosis

Cell apoptosis was quantified using the Annexin V-FITC/PI Apoptosis Detection Kit (Yeasen Biotech, China), following the manufacturer’s instructions. Briefly, harvested cells were washed with PBS and resuspended in 1× binding buffer at a concentration of 1 × 10^6^ cells/mL. The cell suspension was stained with 5 µL each of Annexin V-FITC and propidium iodide (PI), followed by a 15-minute incubation at room temperature in the dark.

Apoptosis was analyzed immediately after staining using a CytoFLEX flow cytometer (Beckman Coulter, USA), with 10,000 events acquired per sample. Apoptotic populations were classified as early apoptotic (Annexin V^+^/PI^-^) and late apoptotic/necrotic (Annexin V^+^/PI^+^). The total apoptotic rate was calculated as the sum of both early and late apoptotic populations.

Data analysis was performed using CytExpert software (Beckman Coulter, USA), and results were expressed as the percentage of apoptotic cells relative to the total population. All values are represented as the mean ± standard deviation (SD) from at least three independent replicates.

### 2.22 Statistical analysis

Statistical analyses were performed using R software (v4.1.3, R Foundation) for biological data processing and GraphPad Prism (v8.0, GraphPad Software) for experimental data analysis. Data from at least three independent replicates are expressed as mean ± SD.

For two-group comparisons, statistical significance was assessed using Student’s t-test. Multi-group comparisons were analyzed by one-way ANOVA followed by Tukey’s *post-hoc* test for multiple comparisons. Significance levels were set at *P < 0.05, **P < 0.01, and ***P < 0.001. All tests were two-tailed, with P < 0.05 considered statistically significant.

## 3 Results

### 3.1 Cellular heterogeneity in glioma progression

To investigate the cellular composition of gliomas, we performed single-nucleus RNA sequencing (snRNA-seq) on tumor samples obtained from seven glioma patients. After rigorous quality control and filtering, 19,232 high-quality cells were selected for further analysis. Through dimensionality reduction and clustering, 21 distinct clusters were identified, which were subsequently categorized into eight major cell types: oligodendrocytes (1,639 cells), astrocytes (2,982 cells), microglia (3,578 cells), smooth muscle cells (332 cells), endothelial cells (257 cells), T cells (135 cells), glioma cells (8,262 cells), and myeloid cells (2,047 cells).

**Figure 1:**
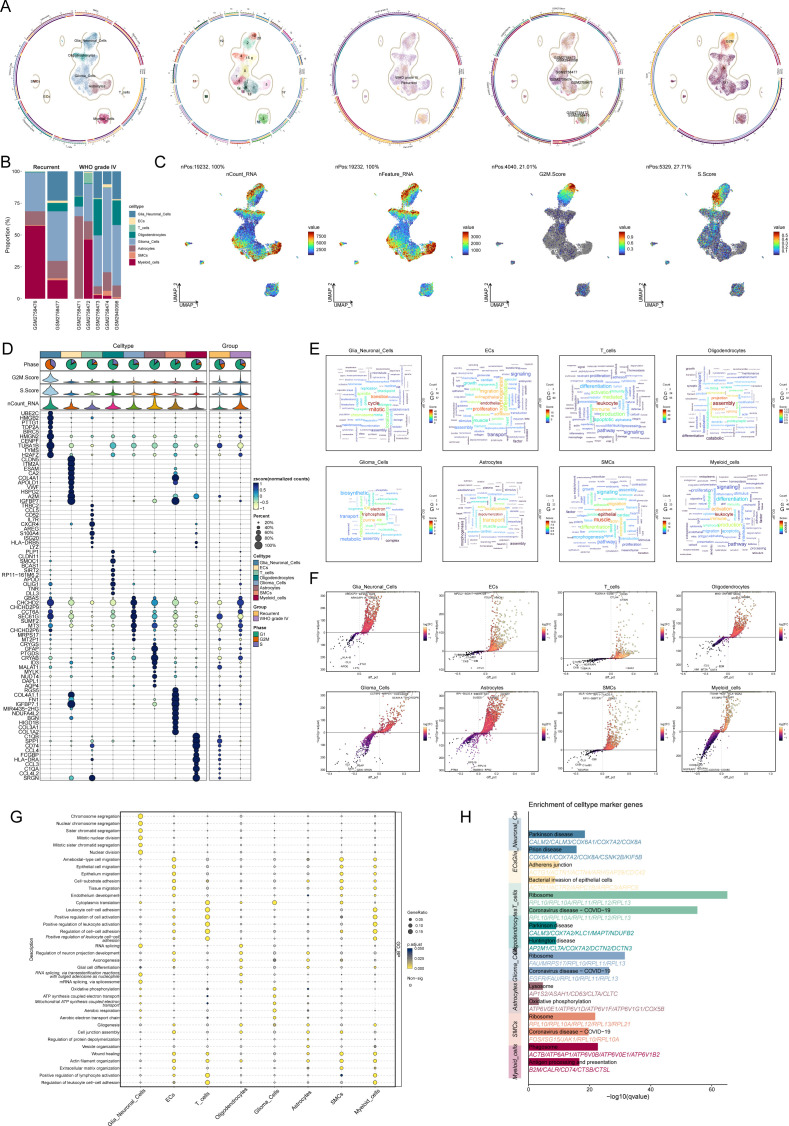
Cellular diversity and gene expression analysis in glioma progression. **(A)** UMAP visualization of cell cycle distribution across glioma samples from seven patients, showing cells in the S phase (3,912 cells), G1 phase (12,498 cells), and G2M phase (2,822 cells). **(B)** Bar plot depicting the relative proportions of eight major cell types (oligodendrocytes, astrocytes, microglia, smooth muscle cells, endothelial cells, T cells, glioma cells, and myeloid cells) in glioma samples from five WHO grade IV glioma patients and two recurrent glioma patients. **(C)** UMAP plot illustrating the distribution of all cells based on nCount_RNA, nFeature_RNA, G2M score, and S score. **(D)** Bubble chart showing the top ten cell type-specific marker genes for each of the eight major cell types identified by snRNA-seq. **(E)** Word cloud highlighting the enriched Gene Ontology (GO) biological process (GO-BP) terms for each of the eight major cell types. **(F)** Volcano plot displaying differentially expressed genes (DEGs) across the eight major cell types. **(G)** Bubble chart depicting the GO-BP enrichment analysis of DEGs across the eight cell types. **(H)** Pathway enrichment analysis of the glioma-associated microglia subgroup and glioma cell subgroup, revealing significant enrichment in pathways related to Parkinson’s disease in microglia and ribosomal function and coronavirus diseases in glioma cells.

The dataset included 5,011 cells from recurrent gliomas and 14,221 cells from WHO grade IV gliomas. UMAP analysis revealed the distribution of these cell types across various cell cycle phases: S phase (3,912 cells), G1 phase (12,498 cells), and G2M phase (2,822 cells) (**Figure 1A**). A bar plot (**Figure 1B**) depicts the proportional distribution of these cell types across samples from five grade IV glioma patients and two recurrent glioma patients.

UMAP plots (**Figure 1C**) were generated to visualize the cell distributions according to nCount_RNA, nFeature_RNA, G2M score, and S score. Analysis of cell type-specific marker gene expression revealed distinct profiles for each cell type, with the top ten marker genes for each type displayed in a bubble chart (**Figure 1D**).

A word cloud (**Figure 1E**) highlights the enriched Gene Ontology (GO) biological process (GO-BP) terms for each cell type. Differentially expressed genes (DEGs) were visualized in volcano plots (**Figure 1F**), and a bubble chart (**Figure 1G**) illustrates the GO-BP enrichment analysis of DEGs across the cell types. Notably, glioma-associated microglia showed enrichment in pathways related to Parkinson’s disease, while glioma cells were primarily associated with pathways involved in ribosomal function and coronavirus-related diseases (**Figure 1H**).

### 3.2 Intratumoral heterogeneity of glioma cells

**Figure 2:**
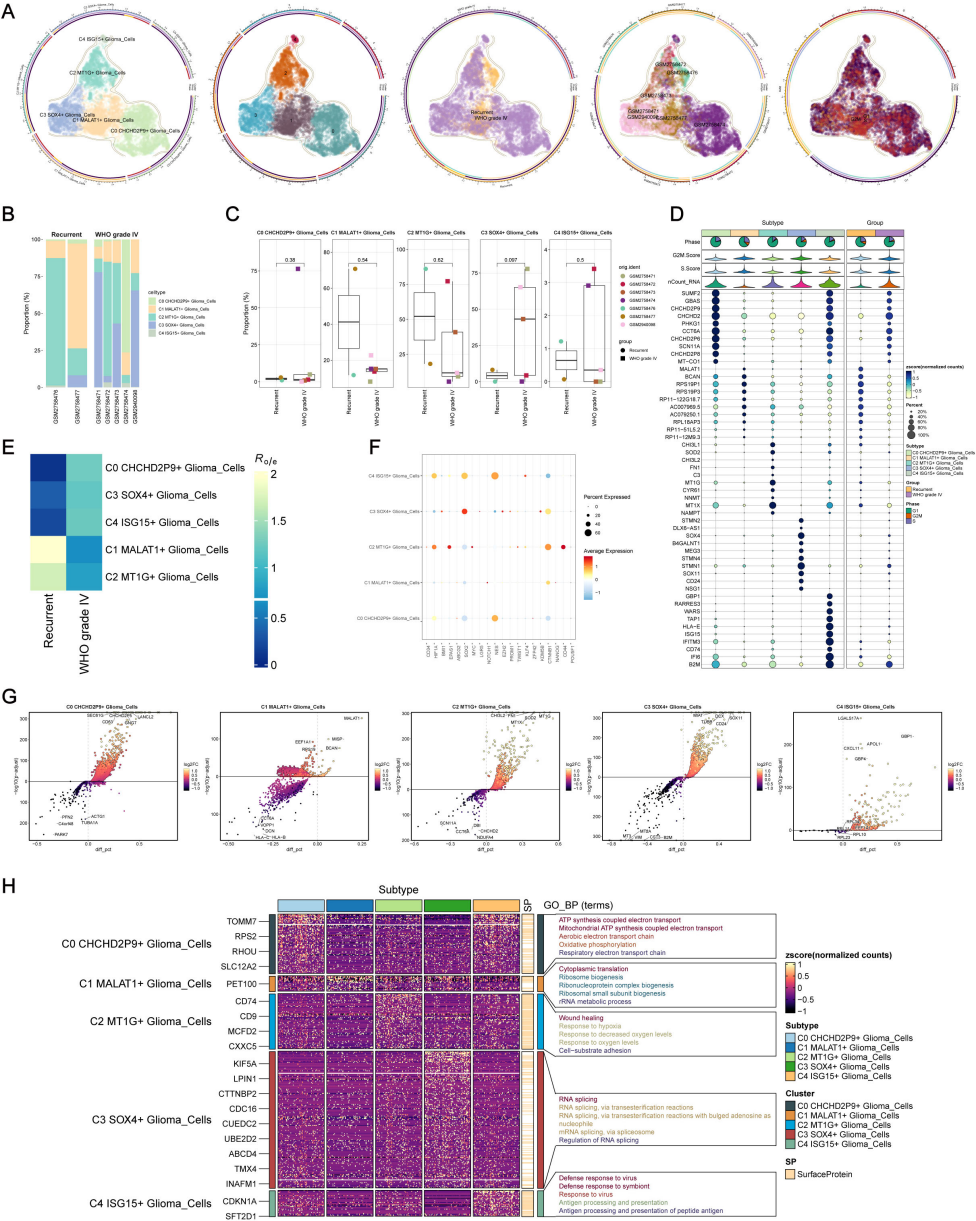
Intracellular heterogeneity of glioma cells. **(A)** Identification of five distinct glioma subpopulations based on dimensionality reduction and clustering analysis: C0 CHCHD2P9+ (2,821 cells), C1 MALT1+ (2,124 cells), C2 MT1G+ (1,614 cells), C3 SOX4+ (1,575 cells), and C4 ISG15+ (128 cells). **(B)** Bar plot showing the distribution of the five glioma subpopulations across samples from five WHO grade IV glioma patients and two recurrent glioma patients. **(C)** Box plot illustrating variations in the proportions of the five glioma subpopulations across different glioma samples. **(D)** Bubble chart visualizing differential expression of marker genes across the five glioma subpopulations. **(E)** Heatmap depicting the specific marker genes for each glioma subpopulation. **(F)** Heatmap showing the enrichment of glioma subpopulations in WHO grade IV versus recurrent gliomas. **(G)** Volcano plot highlighting differentially expressed genes (DEGs) within each glioma subpopulation. **(H)** Heatmap of Gene Ontology (GO) biological process (GO-BP) enrichment analysis for the DEGs across the five glioma subpopulations.

Through clustering and dimensionality reduction techniques, five distinct subpopulations within glioma cells were identified. To distinguish malignant from normal cells in glioblastoma (GBM), the inferCNV algorithm was applied to assess genomic copy number variations (CNVs) at the single-cell level. Cells exhibiting elevated CNV levels were classified as GBM cells (**Supplementary Figure 1**). The five glioma subpopulations were as follows: C0 CHCHD2P9+ glioma cells (2,821 cells), C1 MALT1+ glioma cells (2,124 cells), C2 MT1G+ glioma cells (1,614 cells), C3 SOX4+ glioma cells (1,575 cells), and C4 ISG15+ glioma cells (128 cells) (**Figure 2A**).

To visualize the distribution of these subpopulations, a bar plot was generated from the samples of five grade IV glioma patients and two recurrent glioma patients (**Figure 2B**). The C0 CHCHD2P9+ glioma subpopulation was predominantly present in the GSM2758474 patient. Variability in subpopulation proportions across samples was further depicted using a box plot (**Figure 2C**).

Differential expression of marker genes across the five glioma subpopulations was visualized in a bubble chart (**Figure 2D**), and a heatmap illustrated the specific marker genes for each subpopulation. A separate heatmap was generated to assess the enrichment of these subpopulations in WHO grade IV versus recurrent gliomas (**Figure 2E**). Expression levels of stemness-related genes were also evaluated across the five glioma subpopulations (**Figure 2F**).

Volcano plots were used to highlight the DEGs specific to each glioma subpopulation (**Figure 2G**), while a heatmap was generated to display the results of Gene Ontology Biological Process (GO-BP) enrichment analysis for these DEGs (**Figure 2H**).

### 3.3 Visualization of glioma cell subpopulations via cell tracking and pseudo-temporal analysis

**Figure 3:**
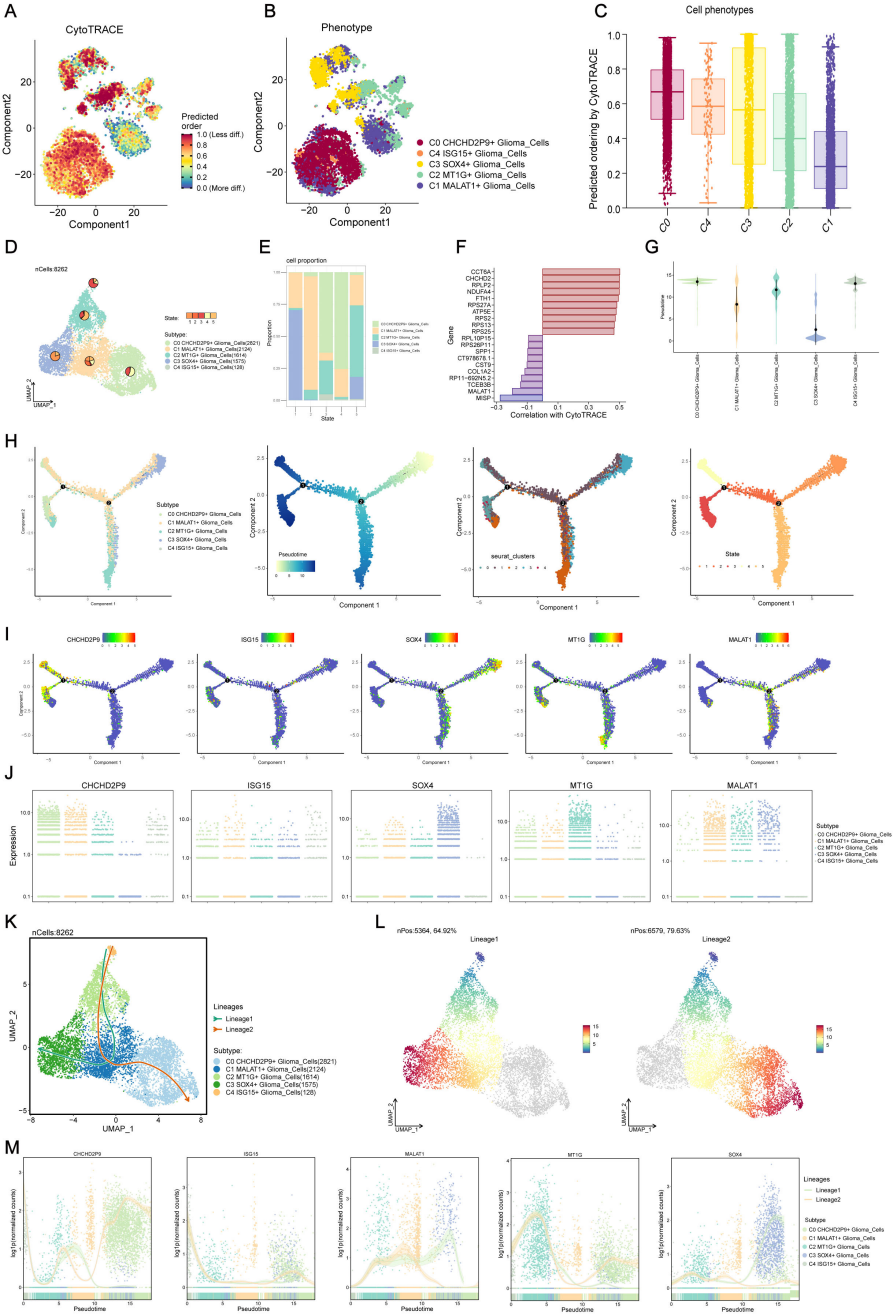
Visualization and Slingshot analysis of glioma cell subpopulations via cell tracking and pseudo-temporal trajectories. **(A, B)** Cell trajectory analysis showing the differentiation relationships among the five glioma subpopulations, revealing the progression from the C1 subpopulation to C2, C3, C4, and ultimately C0. **(C)** Differentiation progression of glioma subpopulations, illustrating the transition from C1 to C0, with C2, C3, and C4 as intermediate states. **(D)** UMAP plot displaying the distribution of cells from each subpopulation across various differentiation states. **(E)** Bar plot showing the percentage distribution of cells within each glioma subpopulation across the different differentiation states identified in **(D)**. **(F)** Visualization of key genes associated with CytoTRACE across the glioma subpopulations, indicating their expression patterns. **(G)** Ridge plot representing the pseudo-temporal ordering of glioma subpopulations along the trajectory. **(H)** Monoclonal trajectory analysis tracing the developmental origins of glioma subpopulations, revealing two distinct branches: Lineage 1, progressing from C4 to C2, C1, and C3, and Lineage 2, progressing from C4 to C2, C1, and converging at C0. **(I)** UMAP plot visualizing the expression patterns of key marker genes for the five glioma subpopulations along the pseudo-temporal trajectory. **(J)** Scatter plot depicting the expression patterns of key marker genes for the five glioma subpopulations along the entire pseudo-temporal sequence, highlighting the C3 SOX4+ subpopulation at the initiation point and the C0 CHCHD2P9+ and C2 MT1G+ subpopulations at the terminal end. **(K)** Slingshot analysis revealing two distinct lineage pathways originating from the C4 ISG15+ glioma subpopulation: Lineage 1 progressing towards C3 and Lineage 2 converging at C0. **(L)** Visualization of differentiation trajectories along the pseudo-temporal axis, showing the bifurcation of Lineage 1 and Lineage 2 towards the C0 and C3 subpopulations, respectively. **(M)** Scatter plot depicting the distribution of glioma subpopulations along the entire pseudo-temporal sequence, illustrating their differentiation along Lineage 1 and Lineage 2 pathways.

To explore the differentiation and developmental dynamics among the five glioma cell subpopulations, a cell trajectory analysis was performed (**Figures 3A, B**). The analysis revealed a differentiation pathway beginning with the C1 subpopulation, progressing through C2, C3, and C4, and ultimately culminating in the C0 subpopulation (**Figure 3C**).

A ridge plot (**Figure 3G**) was used to represent the pseudo-temporal ordering of these subpopulations. UMAP analysis displayed the distribution of cells from each subpopulation across various differentiation states (**Figure 3D**), and a bar plot showed the percentage distribution of cells within each subpopulation across these states (**Figure 3E**). Key genes associated with CytoTRACE were identified and visualized (**Figure 3F**).

Monoclonal trajectory analysis further revealed two distinct branches within the pseudo-temporal sequence. The first branch led towards State 5, while the second branch extended towards State 2, which further bifurcated into two sub-branches, one leading to State 4 and the other to State 3 (**Figure 3H**).

This temporal ordering indicated that the C3 SOX4+ glioma subpopulation is associated with early tumorigenesis, differentiating into other subpopulations as tumor progression occurs. Ultimately, C0 CHCHD2P9+ and C2 MT1G+ subpopulations are formed. Expression patterns of key marker genes for the five subpopulations were visualized along the pseudo-temporal sequence using UMAP and scatter plots (**Figures 3I, J**), demonstrating that the C3 SOX4+ subpopulation predominantly occupies the initiation point of the trajectory, while the C0 CHCHD2P9+ and C2 MT1G+ subpopulations are located at the terminal end.

### 3.4 Slingshot analysis of glioma cell subpopulation pseudo-temporal trajectories

Slingshot analysis was employed to investigate the differentiation pathways of the glioma cell subpopulations. This analysis identified two distinct lineage pathways originating from the C4 subpopulation. In the first lineage (Lineage 1), the path progresses from C4 to C2, C1, and ultimately to C3. The second lineage (Lineage 2) follows a similar progression but converges at C0 (**Figure 3K**). These differentiation patterns were visualized along the pseudo-temporal axis, showing a continuous distribution towards both the C0 and C3 subpopulations, reflecting the bifurcation of the two lineages (**Figure 3L**). Scatter plots further illustrated the distribution of subpopulations along the entire pseudo-temporal sequence, clearly highlighting their differentiation along both Lineage 1 and Lineage 2 (**Figure 3M**).

### 3.5 Cell-cell communication and PDGF signaling pathway analysis using CellChat

**Figure 4:**
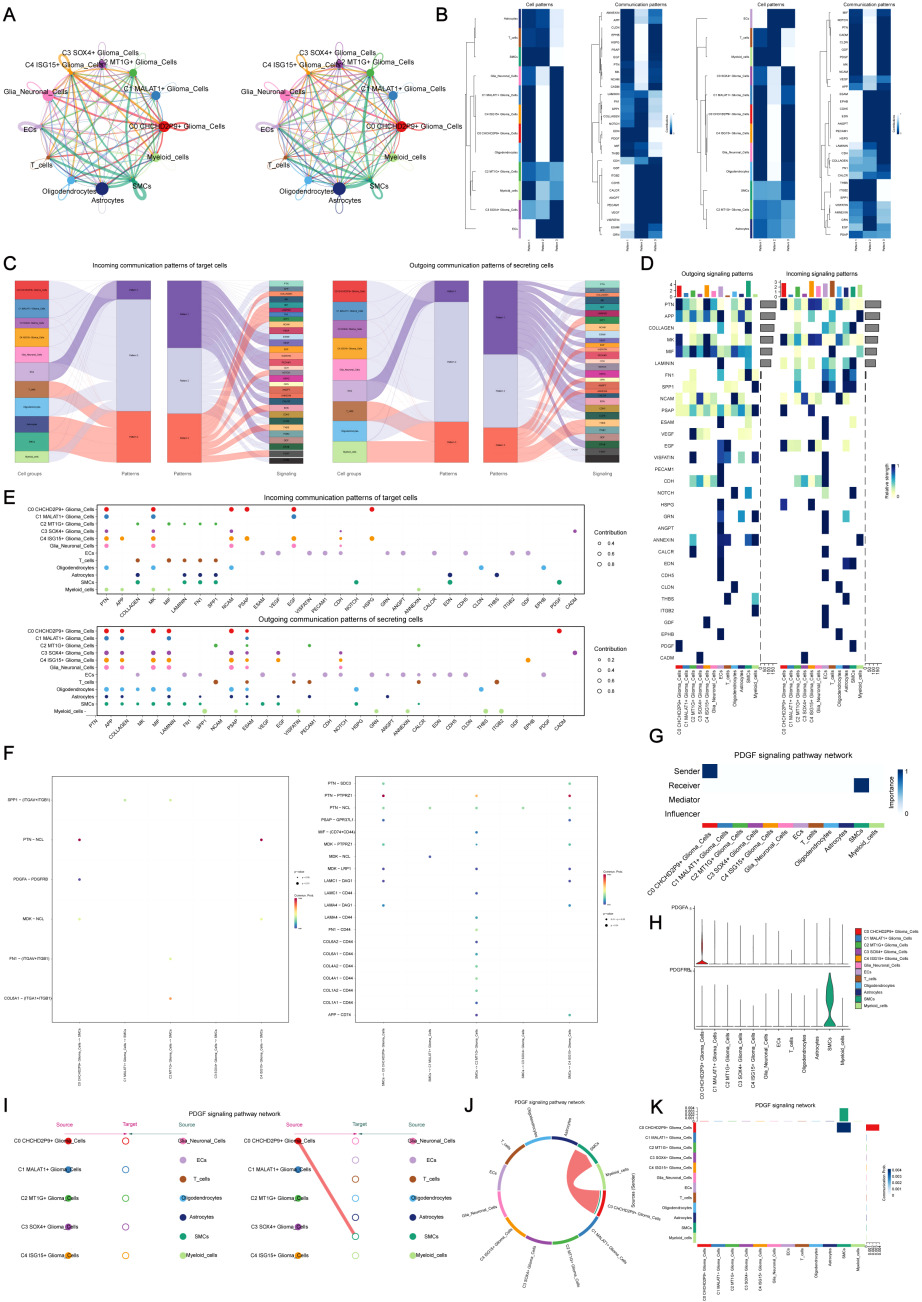
Cell-cell communication and PDGF signaling pathway analysis using CellChat. **(A)** Communication network visualization showing intercellular interactions among glioma subpopulations, oligodendrocytes, myeloid cells, astrocytes, smooth muscle cells (SMCs), and endothelial cells. Line thickness represents interaction strength, and weight represents the quantity of interactions. **(B)** Major signaling patterns identified by CellChat using non-negative matrix factorization, revealing three primary pathways: (1) outgoing communication from endothelial cells, (2) signaling from glioma subpopulations, and (3) signaling from oligodendrocytes and myeloid cells. **(C)** Glioma signaling driven by Pattern 2, highlighting key pathways such as PSAP and PDGF, with incoming signals involving VEGF, NCAM, and PDGF. **(D)** Heatmap visualizing the interaction strength of all signaling pathways within the glioma communication network. **(E)** Ligand-receptor network analysis to identify key signaling molecules involved in intercellular communication within glioma subpopulations. **(F)** Dot plot analysis showing interactions between glioma cells and smooth muscle cells (SMCs), highlighting key communication events. **(G)** Centrality analysis identifying the C0 CHCHD2P9+ glioma subpopulation as central to PDGF signaling. **(H)** Violin plots showing elevated expression of PDGF signaling components in C0 CHCHD2P9+ glioma cells, with high PDGFRB expression in SMCs, suggesting significant interactions between these cell types. **(I)** Hierarchical diagram illustrating the interactions within the PDGF signaling pathway, confirming the association between C0 CHCHD2P9+ glioma cells and SMCs. **(J)** Circular plot depicting PDGF signaling across all twelve cell types, showing the global communication landscape in glioma. **(K)** Heatmap providing detailed insights into the interactions involved in PDGF signaling across the glioma cell types.

CellChat was utilized to explore intercellular communication within gliomas by mapping a network of interactions among various cell types, including glioma subpopulations, oligodendrocytes, myeloid cells, astrocytes, smooth muscle cells (SMCs), and endothelial cells. The intensity and frequency of these interactions were visualized through line thickness and weight, respectively (**Figure 4A**).

The non-negative matrix factorization approach in CellChat identified three primary signaling patterns: (1) communication initiated by endothelial cells, (2) glioma subpopulation-driven signaling, and (3) interactions involving oligodendrocytes and myeloid cells (**Figure 4B**). Glioma signaling was primarily associated with Pattern 2, encompassing pathways such as PSAP and PDGF, while incoming signals were characterized by VEGF, NCAM, and PDGF (**Figure 4C**).

Heatmaps were created to visualize the strength of interactions across all signaling pathways (**Figure 4D**), and ligand-receptor networks were analyzed to identify key signals within glioma subpopulations (**Figure 4E**). Interaction analysis between glioma cells and SMCs was further explored using dot plots (**Figure 4F**).

Centrality analysis revealed that the C0 CHCHD2P9+ glioma subpopulation plays a central role in PDGF signaling (**Figure 4G**). Violin plots demonstrated increased expression of PDGF signaling components in C0 CHCHD2P9+ glioma cells, while SMCs exhibited high PDGFRB expression, suggesting a significant interaction between these cell types (**Figure 4H**). A hierarchical diagram confirmed the association between C0 CHCHD2P9+ glioma cells and SMCs in the PDGF signaling pathway (**Figure 4I**). Finally, a circular plot illustrated PDGF signaling across all twelve cell types (**Figure 4J**), complemented by a heatmap offering further insights into these interactions (**Figure 4K**).

### 3.6 Development and assessment of the predictive model

**Figure 5:**
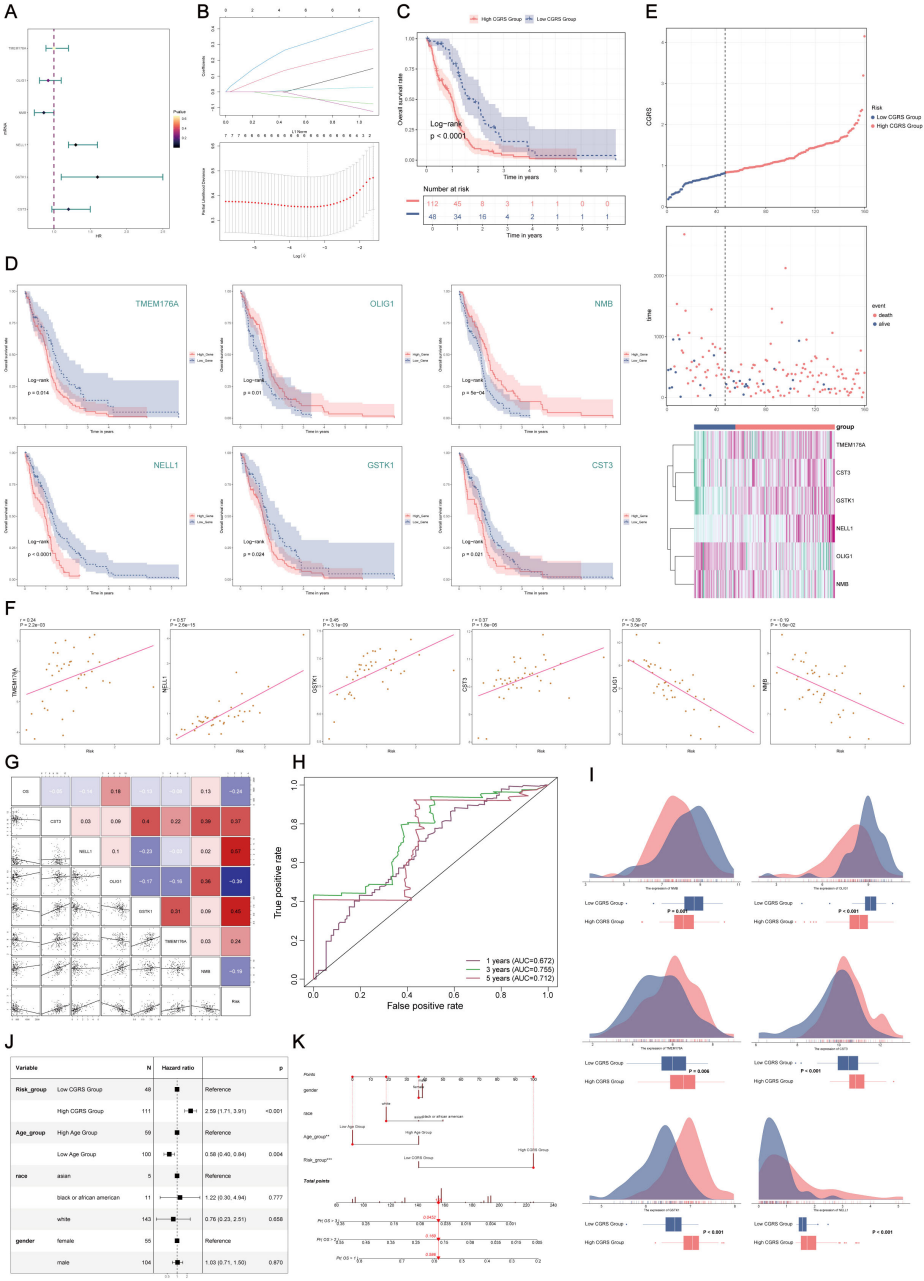
Construction and validation of the prognostic model for C0 CHCHD2P9+ glioma subpopulation. **(A)** Univariate Cox regression analysis of the top 100 marker genes of the C0 CHCHD2P9+ glioma subpopulation, identifying six genes (TMEM176A, OLIG1, NMB, NELL1, GSTK1, and CST3) significantly associated with patient prognosis. **(B)** Lasso regression analysis applied to the identified genes, resulting in the six-gene signature, CHCHD2P9+ Glioma Score, validated through a lambda plot. **(C)** Survival analysis demonstrating that patients with a low CHCHD2P9+ glioma score have significantly better outcomes than those with a high score. **(D)** Survival analysis of individual genes showing that higher expression of OLIG1 and NMB correlates with improved survival, while lower expression of the other genes is associated with worse prognosis. **(E)** Inverse correlation between CHCHD2P9+ glioma score and survival, with higher scores linked to poorer outcomes. **(F)** Scatter plots visualizing the gene expression levels of the six prognostic genes in the CHCHD2P9+ glioma score. **(G)** Correlation analysis showing that overall survival (OS) is positively correlated with OLIG1 and NMB expression, while the other genes show a negative correlation with OS. **(H)** ROC curves evaluating the predictive accuracy of the CHCHD2P9+ glioma score for 1-year, 3-year, and 5-year survival, with AUC values of 0.672, 0.755, and 0.712, respectively. **(I)** Box plot illustrating differences in gene expression between high and low CHCHD2P9+ glioma score groups. **(J)** Multivariate Cox regression analysis confirming that the CHCHD2P9+ glioma score is an independent prognostic factor for glioma patients (p < 0.001). **(K)** Nomogram incorporating gender, race, risk grouping, and age to predict 1-year, 2-year, and 3-year survival probabilities for glioma patients.

Univariate survival analysis of characteristic genes from the C0 CHCHD2P9+ subpopulation identified six prognostic markers—TMEM176A, OLIG1, NMB, NELL1, GSTK1, and CST3—which were significantly associated with patient prognosis (**Figure 5A**). To optimize model performance and reduce feature redundancy, Lasso regularization was applied, resulting in a refined six-gene prognostic signature. Model validation through penalty parameter analysis confirmed the robustness of the selected features (**Figure 5B**).

Stratification of patients based on the developed risk score revealed significant survival differences, with low-risk patients exhibiting better clinical outcomes compared to high-risk patients (**Figure 5C**). Individual gene analysis demonstrated that higher expression of OLIG1 and NMB correlated with favorable prognosis, whereas elevated expression of the remaining markers was associated with poor prognosis (**Figure 5D**). The composite risk score showed a strong inverse relationship with patient survival, with higher scores predicting progressively worse outcomes (**Figure 5E**).

Quantitative expression profiling and correlation analysis confirmed the varying prognostic effects of the individual markers(**Figure 5F**). OLIG1 and NMB expression were positively correlated with overall survival, while the other markers were negatively associated (**Figure 5G**). Time-dependent receiver operating characteristic (ROC) analysis demonstrated strong predictive performance, with area under the curve (AUC) values of 0.672, 0.755, and 0.712 for 1-, 3-, and 5-year survival predictions, respectively (**Figure 5H**). Comparative expression analysis between risk groups further confirmed the model’s ability to effectively discriminate survival outcomes (**Figure 5I**).

Multivariate survival analysis identified the risk score as an independent prognostic factor (p < 0.001) (**Figure 5J**). To facilitate clinical application, a comprehensive predictive tool was developed, incorporating demographic and molecular features to provide personalized survival probability estimates at 1, 2, and 3 years (**Figure 5K**).

### 3.7 Tumor immune microenvironment characterization

**Figure 6:**
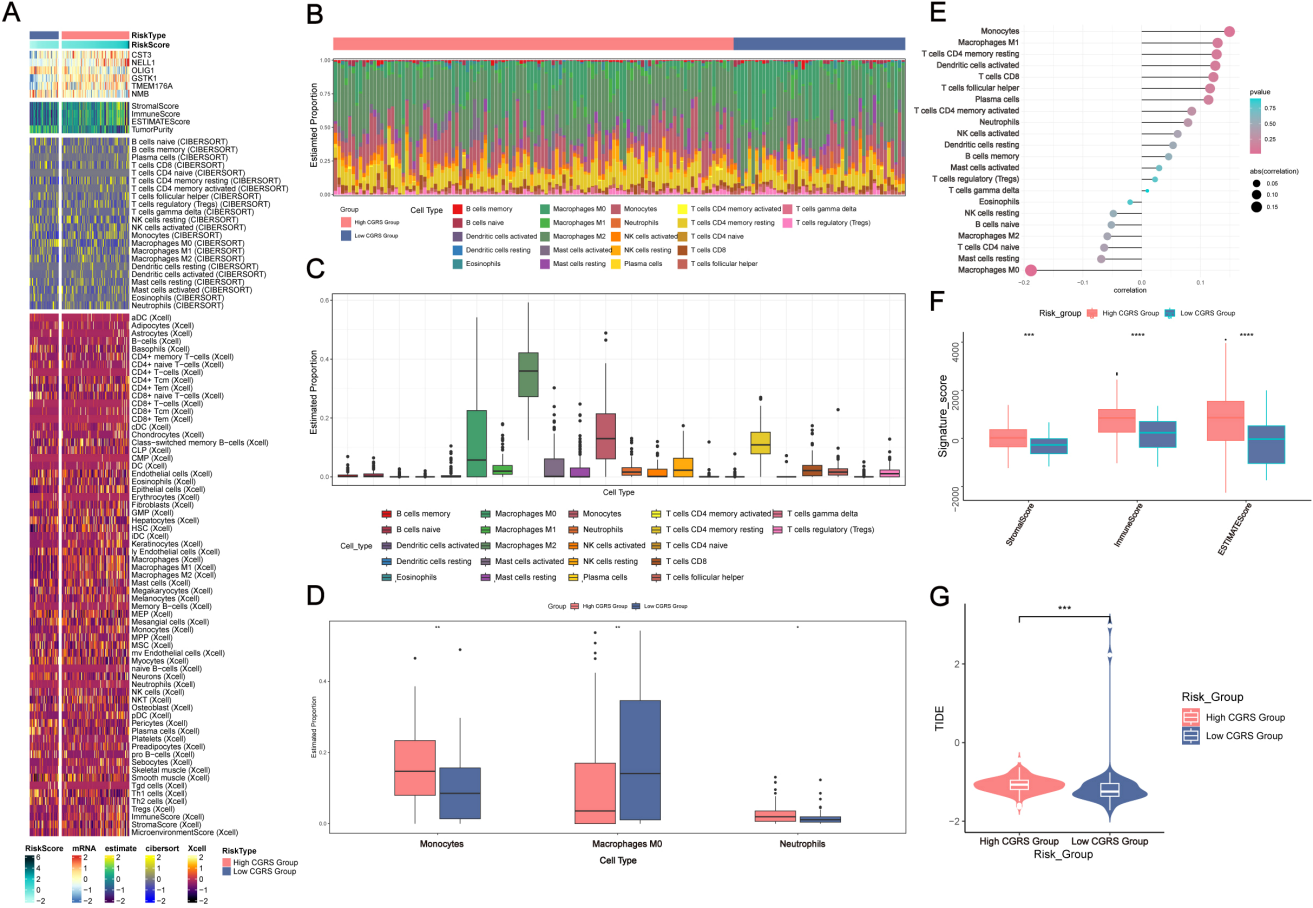
Immune infiltration analysis in glioma based on CHCHD2P9+ glioma score. **(A)** Heatmap showing the differential expression of immune cells between the high and low CHCHD2P9+ glioma score groups. **(B, C)** Proportions of 22 immune cell types in each group, as determined by the CIBERSORT algorithm, highlighting differences in immune cell composition between high and low CHCHD2P9+ glioma score groups. **(D)** Bar plot demonstrating significant differences in immune cell composition, with higher levels of monocytes, M0 macrophages, and neutrophils in the high CHCHD2P9+ glioma score group. **(E)** Lollipop plot illustrating the correlation between immune cell infiltration and glioma subpopulation marker scores. Positive correlations are observed with monocytes and M1 macrophages, while negative correlations are seen with M0 macrophages, resting mast cells, and several other immune cell types. **(F)** Comparison of stromal, immune, and ESTIMATE scores between the high and low CHCHD2P9+ glioma score groups, with significantly higher scores in the high score group. **(G)** Tumor purity analysis showing that the high CHCHD2P9+ glioma score group exhibits higher tumor purity compared to the low score group. *P<0.05, **P<0.01, *** P < 0.001, and ****P<0.0001.

Comparative analysis of immune cell distribution patterns across glioma subgroups, stratified by CHCHD2P9 expression levels, was performed using hierarchical clustering (**Figure 6A**). Quantitative assessment of tumor-infiltrating immune populations through computational deconvolution revealed distinct immune cell proportions among 22 subtypes in each expression subgroup (**Figures 6B, C**). The high-expression cohort demonstrated elevated infiltration of specific myeloid populations, including monocytic cells, non-polarized macrophages, and neutrophilic granulocytes (**Figure 6D**).

Bivariate correlation analysis using hybrid visualizations showed significant associations between immune infiltration patterns and glioma subpopulation characteristics. CHCHD2P9 expression levels were positively correlated with monocyte-derived cells and classically activated macrophages, while negatively associated with non-activated macrophages, quiescent mast cells, and other immune subsets (**Figure 6E**).

Quantitative evaluation of tumor microenvironment components revealed significant differences between expression subgroups. The high-expression cohort exhibited elevated composite scores reflecting stromal content, immune infiltration, and overall tumor microenvironment complexity. Tumor cellularity assessment indicated a greater proportion of neoplastic cells in samples with elevated CHCHD2P9 expression compared to those with low expression (**Figures 6F, G**).

### 3.8 Differential gene expression and functional enrichment analysis

**Figure 7:**
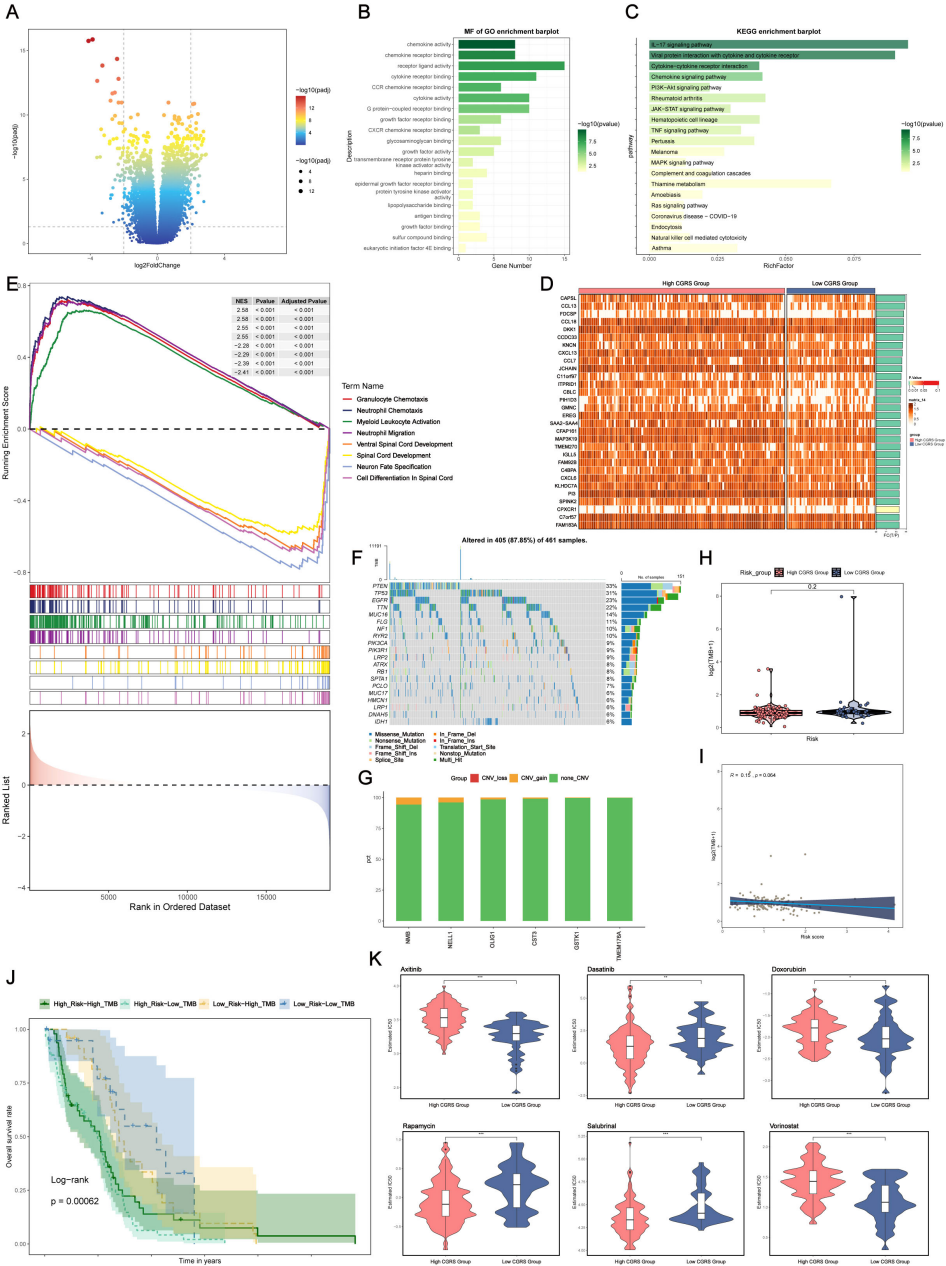
Differential gene expression, functional enrichment, mutation analysis, and drug sensitivity in glioma based on CHCHD2P9+ glioma score. **(A)** Volcano plot showing differential gene expression between high and low CHCHD2P9+ glioma score groups. **(B)** Gene Ontology (GO) enrichment analysis of differentially expressed genes, highlighting significant associations with chemokine activity, receptor-ligand interactions, and chemokine receptor binding. **(C)** Kyoto Encyclopedia of Genes and Genomes (KEGG) pathway analysis showing significant enrichment of genes in pathways related to interleukin-17 signaling, viral protein-cytokine interactions, and cytokine-receptor interactions. **(D)** Heatmap visualizing gene expression patterns between high and low CHCHD2P9+ glioma score groups. **(E)** Gene Set Enrichment Analysis (GSEA) of GO biological processes (GO-BP), highlighting enriched biological pathways. **(F)** Mutation analysis showing the top 20 genes with the highest mutation frequencies in mesenchymal cells across both high and low CHCHD2P9+ glioma score groups. The upper panel displays mutation burden for each sample, while the right panel shows overall mutation proportions. **(G)** Chromosomal copy number variation (CNV) analysis, presented in a bar graph, revealing a slight increase in CNVs for model genes, with no significant decrease. **(H)** Violin plot comparing mutation burden between high and low CHCHD2P9+ glioma score groups, with no significant differences observed. **(I)** Scatter plot showing the correlation between mutation burden and CHCHD2P9+ glioma scores, with no significant association. **(J)** Survival analysis based on mutation burden and CHCHD2P9+ glioma scores, revealing that the low CHCHD2P9+ glioma score–low TMB group had the highest survival rate, while the high CHCHD2P9+ glioma score–low TMB group had the poorest survival rate. **(K)** Violin plot showing drug sensitivity profiles, with significantly lower IC50 values for dasatinib in the high CHCHD2P9+ glioma score group, indicating increased sensitivity. *P<0.05, **P<0.01, *** P < 0.001.

To investigate the variations in gene expression between glioma samples with high and low CHCHD2P9+ scores, differential expression was assessed through a volcano plot (**Figure 7A**), and gene expression patterns were visualized in a heatmap (**Figure 7D**).

Functional enrichment analysis of differentially expressed genes (DEGs) revealed that these genes were predominantly associated with chemokine activity, receptor-ligand interactions, and chemokine receptor binding (**Figure 7B**), suggesting their involvement in inflammatory responses and immune modulation within the tumor microenvironment.

KEGG pathway analysis identified significant enrichment in pathways related to interleukin-17 signaling, viral protein-cytokine interactions, and cytokine-receptor interactions (**Figure 7C**), highlighting key immune-related signaling pathways that may contribute to glioma progression. Additionally, Gene Set Enrichment Analysis (GSEA) of Gene Ontology (GO) biological processes (GO-BP) further confirmed the enrichment of these genes in biologically significant pathways related to immune responses and cell signaling (**Figure 7E**).

### 3.9 Genomic alteration profiling

To examine potential relationships between genomic alterations and the immune landscape, we conducted a comparative mutation analysis across glioma subgroups stratified by CHCHD2P9 expression levels. This analysis focused on the most frequently mutated genes in mesenchymal cell populations, with the upper panel depicting sample-specific mutation profiles, and the right panel showing aggregate mutation frequencies across the cohort (**Figure 7F**).

Genomic instability was assessed through chromosomal aberration profiling, presented as a quantitative bar graph, revealing moderate amplification events in model genes, without substantial genomic loss (**Figure 7G**). A comparative analysis of genomic alteration frequencies between subgroups with high and low CHCHD2P9 expression, visualized through density distribution plots, indicated no statistically significant differences in mutation rates (**Figure 7H**). Furthermore, correlation analysis between the genomic alteration load and CHCHD2P9 expression levels revealed no significant association (**Figure 7I**).

Classification of tumor samples based on genomic alteration load and CHCHD2P9 expression levels identified four distinct subgroups. Kaplan-Meier survival analysis demonstrated significant prognostic differences between these subgroups, with patients exhibiting low CHCHD2P9 expression and minimal genomic alterations having the most favorable clinical outcomes. Conversely, cases with high CHCHD2P9 expression and low genomic alteration burden were associated with the poorest survival rates (**Figure 7J**).

### 3.10 Drug sensitivity analysis

To evaluate variations in drug sensitivity between glioma groups with high and low CHCHD2P9+ scores, drug sensitivity profiles were generated and visualized using a violin plot (**Figure 7K**). The analysis revealed that the high CHCHD2P9+ score group exhibited a significantly lower IC50 value for dasatinib, indicating an increased sensitivity to this drug compared to the low score group.

### 3.11 Genetic silencing of CHCHD2P9 inhibits glioma cell growth and motility

Computational analysis identified distinct expression patterns of CHCHD2P9 across different glioma subtypes, challenging its conventional annotation as a non-functional pseudogene. To further elucidate its biological functions, RNA interference technology was employed to specifically silence CHCHD2P9 expression in glioma cell models. Lentiviral-mediated delivery of short hairpin RNAs (shRNAs) successfully generated stable knockdown lines in both LN229 and U87 glioma cells, with silencing efficiency verified by quantitative PCR (**Supplementary Figure 2**).

**Figure 8:**
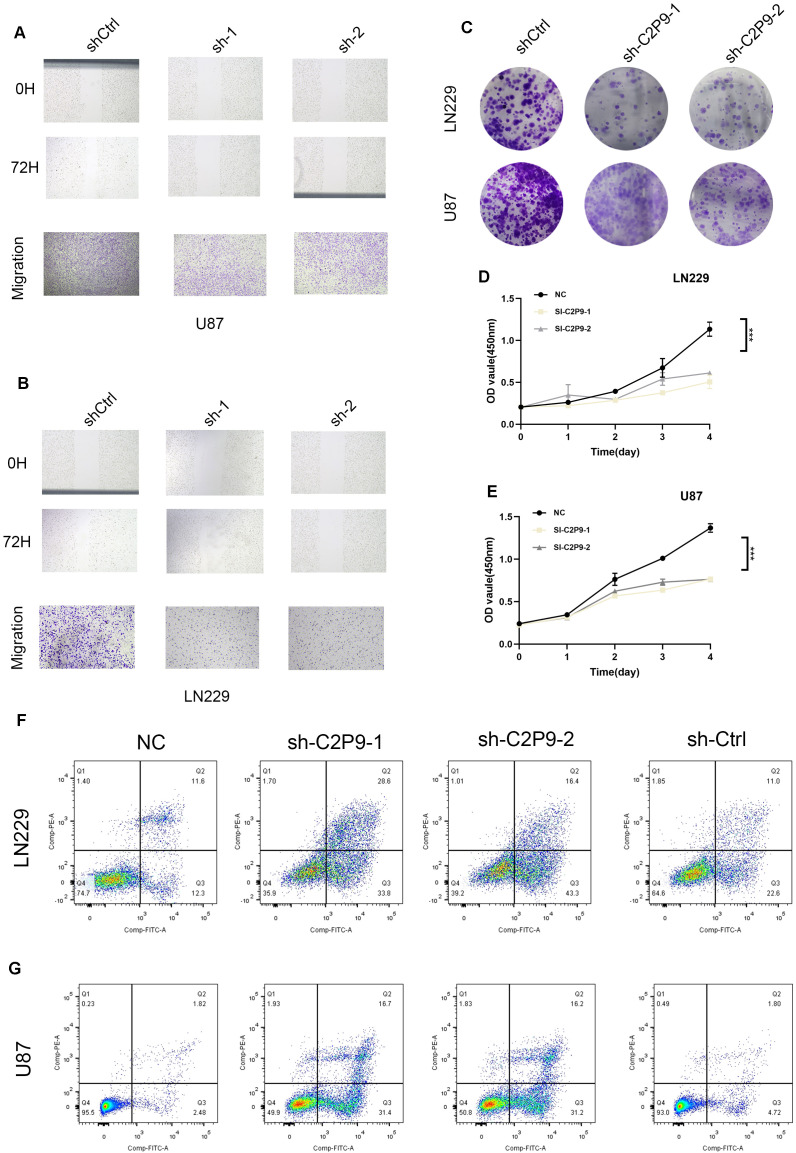
Knockdown of CHCHD2P9 suppresses proliferation, migration, and induces apoptosis in glioma cells. **(A)** Scratch wound healing assay and transwell migration assay demonstrating a significant reduction in wound closure and a marked decrease in the number of migrating in CHCHD2P9 knockdown U87 cells compared to control cells, indicating impaired migratory ability. **(B)** Scratch wound healing assay and transwell migration assay showing a significant reduction in wound closure and a marked decrease in the number of migrating CHCHD2P9 knockdown LN229 cells relative to controls, suggesting reduced migratory capacity. **(C)** Colony formation assay revealing a significant reduction in colony formation in CHCHD2P9 knockdown U87 and LN229 cells, indicating decreased proliferative activity. **(D)** CCK-8 assay showing a significant decrease in absorbance in CHCHD2P9 knockdown LN229 cells, suggesting reduced cell viability. **(E)** CCK-8 assay demonstrating decreased cell viability in CHCHD2P9 knockdown U87 cells compared to controls, confirming proliferative impairment. **(F, G)** Flow cytometry analysis showing a significant increase in the apoptosis rate in CHCHD2P9 knockdown U87 and LN229 cells compared to controls, indicating that CHCHD2P9 knockdown induces apoptosis in glioma cells. *** P < 0.001.

Wound closure assays demonstrated significantly impaired migration in CHCHD2P9-deficient U87 cells compared to their wild-type counterparts, indicating a critical role for CHCHD2P9 in promoting glioma cell motility. This result was further corroborated by Transwell migration assays, which revealed a substantial decrease in the transmigration capacity of CHCHD2P9-deficient cells (**Figure 8A**). Parallel experiments conducted in LN229 cells yielded similar results, suggesting that CHCHD2P9 influences glioma cell migration across different cellular contexts (**Figure 8B**).

Next, the proliferative capacity of glioma cells was assessed through clonogenic survival assays. CHCHD2P9-depleted cells exhibited significantly reduced colony-forming efficiency, suggesting impaired long-term growth potential (**Figure 8C**). Cellular metabolic activity, measured by CCK-8 assays, showed a corresponding decrease in both LN229 and U87 cells following CHCHD2P9 silencing, indicating compromised cell viability (**Figures 8D, E**).

To investigate the mechanisms underlying these phenotypic changes, we assessed apoptosis using flow cytometry. CHCHD2P9-deficient cells exhibited a significantly higher apoptotic index compared to both untreated and negative control cells (**Figures 8F, G**), implicating CHCHD2P9 in the regulation of glioma cell survival. These results suggest that CHCHD2P9 may play a critical role in modulating apoptotic pathways and promoting glioma cell survival and motility.

## 4 Discussion

Gliomas, particularly high-grade gliomas, represent a significant clinical challenge due to their aggressive nature, substantial heterogeneity, and high mortality rates. The complexity of neurobiological research presents substantial challenges, especially when investigating gliomas, which are highly aggressive and often fatal brain tumors (29). Despite advances in surgical approaches, radiation therapy, and chemotherapy, the treatment of gliomas remains difficult due to their inherent resistance to conventional therapies, as well as the physical barrier imposed by the blood-brain barrier (BBB) (30). These tumors’ ability to adapt to treatment pressures and their aggressive growth patterns necessitate the identification of novel therapeutic strategies. As research continues to evolve, there is an increasing appreciation of the role that cellular organelles, particularly mitochondria, play in the pathogenesis of gliomas. While the functions of mitochondria in glioma cells remain an active area of investigation, growing evidence suggests that mitochondria are integral in regulating critical cellular processes such as metabolism, apoptosis, and drug resistance. However, the precise mechanisms by which mitochondrial dysfunction contributes to glioma progression remain incompletely understood. This is, in part, due to the complex nature of tumor biology and the challenges inherent in studying dynamic organellar alterations within the tumor microenvironment (TME) (31, 32). Recent advancements in single-cell technologies have provided unprecedented insights into the cellular heterogeneity of gliomas, offering a detailed view of the tumor microenvironment and revealing distinct cellular populations that contribute to glioma progression. Single-cell RNA sequencing (scRNA-seq), for instance, has enabled the identification of previously unrecognized cellular subsets within gliomas, facilitating a deeper understanding of their functional roles in tumor development and progression (33, 34). This technology is also opening new possibilities for precision medicine, by uncovering the intricate interactions between tumor cells and their surrounding stromal elements, including immune cells and vasculature. Through this approach, we identified the C0 CHCHD2P9+ glioma subpopulation as a critical contributor to glioma advancement, emphasizing the potential of this population as a prognostic biomarker and a therapeutic target. The elevated expression of CHCHD2P9 within this cellular subset suggests its potential role in regulating glioma cell differentiation, lineage commitment, and the maintenance of tumor aggressiveness (35). These technologies are anticipated to expedite the elucidation of glioma pathogenesis and play a role in the development of more targeted and efficacious therapies.

Our findings revealed that the C0 CHCHD2P9+ population predominantly occupies terminal differentiation states, which correlates with its involvement in advanced tumor stages (14). Pseudo-temporal reconstruction and trajectory inference analyses further supported the notion that these cells serve as pivotal nodes in the differentiation networks of glioma cells. These results are consistent with recent studies suggesting that gliomas exhibit a highly dynamic and heterogeneous differentiation landscape, where certain subpopulations, such as C0 CHCHD2P9+ cells, may drive tumor progression by occupying differentiated states that are resilient to therapy and contribute to tumor recurrence. Furthermore, intercellular communication analysis highlighted the prominent role of this subpopulation in platelet-derived growth factor (PDGF)-mediated signaling, particularly through interactions with vascular smooth muscle cells. Given PDGF’s well-established role in promoting angiogenesis and supporting tumor growth, these findings suggest that CHCHD2P9+ cells might contribute to glioma’s malignant phenotype by facilitating neoplastic expansion and enhancing metastatic potential through increased vascularization (36). The activation of the PDGF pathway in this context reinforces the importance of targeting this signaling axis as a potential therapeutic strategy in glioma management (36, 37).

The association between CHCHD2P9 expression and PDGF signaling underscores the complex interplay between metabolic reprogramming, cell motility, and the immune landscape in glioma progression. Given the critical involvement of PDGF signaling in glioma pathogenesis, therapeutic modulation of this pathway holds significant promise (38, 39). CHCHD2P9+ cells demonstrated associations with PDGF-mediated signaling and displayed enrichment patterns suggesting involvement in immunologically relevant pathways. Single-cell and CIBERSORT analyses revealed that higher CHCHD2P9 expression correlated with features of an immunosuppressive tumor microenvironment, including an increased presence of tumor-associated macrophages and reduced infiltration of cytotoxic immune cells. These observations indicate a potential role for CHCHD2P9 in shaping immune cell dynamics within gliomas; however, causal relationships remain unproven. It is also possible that CHCHD2P9 serves as a biomarker of a more aggressive and immune-evasive glioma subtype, rather than directly driving immune evasion mechanisms. Future studies involving direct immunological assays (e.g., cytokine profiling, co-culture systems) are necessary to clarify these relationships.

Our investigation into the biological functions of CHCHD2P9, a mitochondrial-associated protein, further elucidated its role in glioma cell growth dynamics and motility. Through loss-of-function studies, including RNA interference approaches, we demonstrated that CHCHD2P9 significantly influences glioma cell migration and proliferation. These results align with previous studies highlighting the pivotal role of mitochondrial proteins in regulating cellular motility, a key determinant of glioma invasiveness and metastasis. Mitochondria are central to energy production and cellular signaling, and their dysfunction can impair essential processes such as cell division, migration, and apoptosis. Our findings suggest that CHCHD2P9 is critical for maintaining mitochondrial integrity and cellular motility, which are essential for glioma invasiveness and tumor progression (40). The impairment of migration and clonogenic survival following CHCHD2P9 silencing underscores its role in sustaining glioma cell viability and growth potential. These observations are consistent with the broader understanding that mitochondrial dysfunction plays a key role in tumor cell survival, particularly in the context of highly aggressive malignancies such as gliomas.

Beyond its established role in promoting migration and proliferation, our findings demonstrate that silencing CHCHD2P9 significantly compromises glioma cell viability, highlighting its critical function in sustaining tumor cell survival. Mitochondria are central to cellular energy production, redox balance, and apoptotic regulation, and the observed reduction in cell viability upon CHCHD2P9 knockdown suggests that this gene may play an essential role in maintaining mitochondrial integrity and metabolic homeostasis in glioma cells. Intriguingly, CHCHD2 family members have been previously implicated in the regulation of mitochondrial protein complexes and oxidative phosphorylation. CHCHD2P9 is annotated as a processed pseudogene, and although it lacks protein-coding potential, it may exert regulatory functions through non-coding RNA mechanisms. Similar to other pseudogenes, CHCHD2P9 transcripts may act as competitive endogenous RNAs (ceRNAs), sequestering microRNAs and thereby modulating the expression of target genes, including its parental gene CHCHD2. This mode of action has been observed in various cancers, where pseudogene-derived RNAs influence tumor behavior by affecting signaling cascades, gene expression networks, or cellular stress responses. While our current study does not provide direct evidence for such regulatory interactions, the possibility that CHCHD2P9 functions in this capacity warrants further investigation.

Nonetheless, this study is not without limitations. Our *in vitro* work primarily utilized two classical glioma cell lines, LN229 and U87, which, while informative, may not fully capture the heterogeneity observed in patient-derived tumors. Moreover, while our computational analyses suggest associations between CHCHD2P9 expression and immune-suppressive features in the glioma microenvironment, these findings remain correlative. We acknowledge the lack of direct experimental validation, such as immune cell co-culture or cytokine assays, which are required to substantiate the hypothesized immune modulatory effects. Thus, the proposed links between CHCHD2P9 and immune evasion should be interpreted as speculative and warrant future mechanistic investigation (41, 42).

In conclusion, our study provides compelling evidence that CHCHD2P9 plays a crucial role in regulating glioma cell growth, motility, and survival, with significant implications for glioma pathogenesis. The involvement of CHCHD2P9 in mitochondrial processes positions it as a potential therapeutic target for gliomas, particularly in the context of targeting mitochondrial dysregulation. Future studies should focus on further characterizing the molecular interactions of CHCHD2P9 in glioma cells, exploring its diagnostic potential, and evaluating its therapeutic relevance in clinical settings. In addition, given the potential immune modulatory role of CHCHD2P9+ cells within the glioma TME, targeting this population may offer new strategies for enhancing the efficacy of immunotherapies in glioma treatment.

## 5 Conclusions

In conclusion, our study identifies CHCHD2P9 as a key mitochondrial-related protein involved in glioma progression. Functional assays show that CHCHD2P9 silencing significantly impairs glioma cell proliferation and migration, suggesting its crucial role in tumorigenesis. Notably, its impact extends beyond tumor cell dynamics, potentially influencing the tumor microenvironment through signaling pathways such as PDGF. These findings highlight CHCHD2P9 as a promising therapeutic target in glioma treatment. Future research should focus on elucidating its molecular pathways, particularly its interaction with immune cells in the tumor microenvironment, to develop novel strategies aimed at enhancing immune responses and targeting mitochondrial dysfunction in glioma therapy.

## Data Availability

The original contributions presented in the study are included in the article/**Supplementary Material**. Further inquiries can be directed to the corresponding authors.

## Supplementary material

Supplementary Figure 1 The classification of GBM cells. According to the inferCNV results, we defined cells with high CNV levels as GBM cells.

Supplementary Figure 2 CHCHD2P9 gene transfection knock-down low efficiency verification. Compare d with untransfected cells, the mRNA level of CHCHD2P9 gene was significantly decreased in the transfected knockdown group.
