# Supplementary figures and images for: Molecular insights into glioblastoma progression: role of CHCHD2P9 in tumor heterogeneity and prognosis
Source: Front Immunol. 2025 Jun 24;16:1581850. doi: 10.3389/fimmu.2025.1581850 (PMC12234496; doi:10.3389/fimmu.2025.1581850)

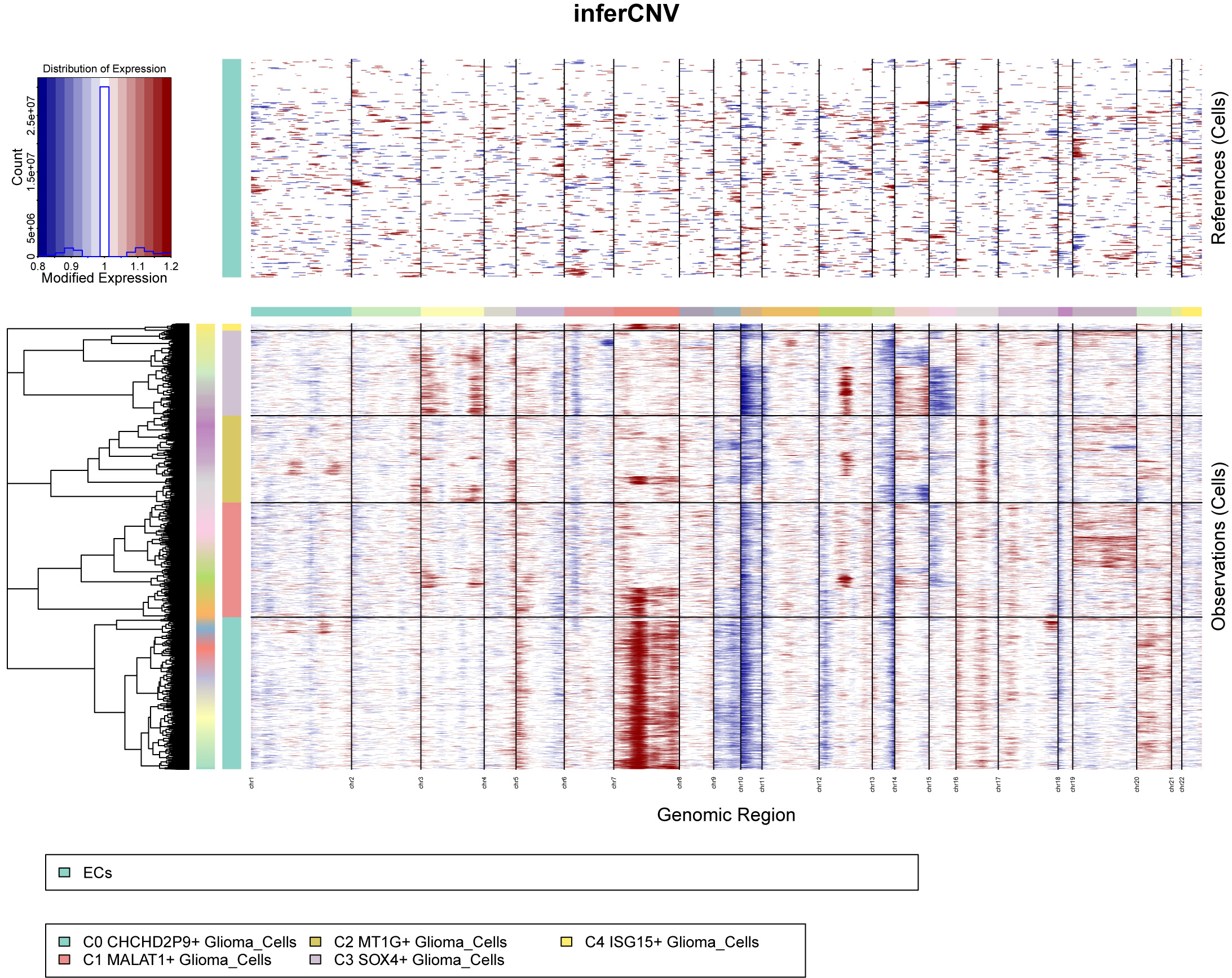

Supplement: Supplementary Figure 1 — The classification of GBM cells. According to the inferCNV results, we defined cells with high CNV levels as GBM cells. [file Image1.jpeg]

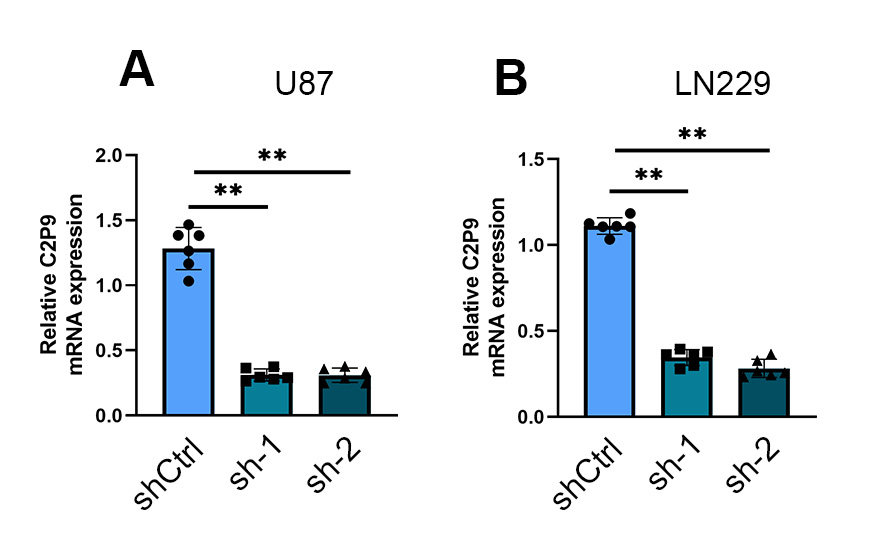

Supplement: Supplementary Figure 2 — CHCHD2P9 gene transfection knock-down low efficiency verification. Compare d with untransfected cells, the mRNA level of CHCHD2P9 gene was significantly decreased in the transfected knockdown group. [file Image2.jpeg]
