# Supplementary material for: Molecular insights into glioblastoma progression: role of CHCHD2P9 in tumor heterogeneity and prognosis
Source: Front Immunol. 2025 Jun 24;16:1581850. doi: 10.3389/fimmu.2025.1581850 (PMC12234496; doi:10.3389/fimmu.2025.1581850)
Supplement: Supplementary file 3 [file Table1.docx]

| **Oligonucleotides** | **Nucleotide sequence (5'-3')** |
| --- | --- |
| **shRNA** |  |
| shRNA-NC | GCUUCGCGCCGUAGUCUUA |
| shC2P9-1 | GATCCGAGGCTGAAGGACCTGAGAGTCAAGAGACTCTCAGGTCCTTCAGCCTCGTTTTT |
| shC2P9-2 | GATCCGAGGAAGCCTGGAGTCAAGAGACTCTCAGGAGGCTCCTCAGGCCTCGTTTTT |
| **Primer** |  |
| β-Actin | **CATGTACGTTGCTATCCAGGC** (forward) |
|  | **CTCCTTAATGTCACGCACGAT** (reverse) |
| C2P9 | CAGGAAGCTTCAGGAGGA (forward) |
|  | TGAAGATGTCATTCAGTG (reverse) |
|  |  |

**Table S1. Oligonucleotides used in research**
